# Central Administration of Angiotensin-(1-7) Improves Vasopressin Impairment and Hypotensive Response in Experimental Endotoxemia

**DOI:** 10.3390/cells10010105

**Published:** 2021-01-08

**Authors:** Patrícia Passaglia, Felipe de Lima Faim, Marcelo Eduardo Batalhão, Angelita Maria Stabile, Lusiane Maria Bendhack, José Antunes-Rodrigues, Riccardo Lacchini, Evelin Capellari Carnio

**Affiliations:** 1 Department of Physiology, Ribeirão Preto Medical School, University of São Paulo, Ribeirão Preto, São Paulo 14049-900, Brazil; ppassaglia@usp.br (P.P.); faim10@gmail.com (F.d.L.F.); antunes@usp.br (J.A.-R.); 2 Department of General and Specialized Nursing, Ribeirão Preto College of Nursing, University of São Paulo, Ribeirão Preto, São Paulo 14040-902, Brazil; batalhao@eerp.usp.br (M.E.B.); angelita@eerp.usp.br (A.M.S.); 3 Department of Physics and Chemistry, Faculty of Pharmaceutical Sciences of Ribeirão Preto, Ribeirão Preto-University of São Paulo, Ribeirão Preto, São Paulo 14040-903, Brazil; bendhack@usp.br; 4 Department of Psychiatric Nursing and Human Science, Ribeirão Preto College of Nursing, University of São Paulo, Ribeirão Preto, São Paulo 14040-902, Brazil; rlacchini@eerp.usp.br

**Keywords:** Angiotensin-(1-7), Mas receptor, endotoxemia, systemic inflammation, vasopressin, vascular reactivity, hypotension

## Abstract

Angiotensin-(1-7) [Ang-(1-7)]/Mas receptor is a counter-regulatory axis that counteracts detrimental renin-angiotensin system (RAS) effects, especially regarding systemic inflammation, vasopressin (AVP) release, and hypothalamic-pituitary-adrenal (HPA) activation. However, it is not completely understood whether this system may control centrally or systemically the late phase of systemic inflammation. Thus, the aim of this study was to determine whether intracerebroventricular (i.c.v.) administration of Ang-(1-7) can modulate systemic inflammation through the activation of humoral pathways in late phase of endotoxemia. Endotoxemia was induced by systemic injection of lipopolysaccharide (LPS) (1.5 mg/kg, i.v.) in Wistar rats. Ang-(1-7) (0.3 nmol in 2 µL) promoted the release of AVP and attenuated interleukin-6 (IL-6) and nitric oxide (NO) levels but increased interleukin-10 (IL-10) in the serum of the endotoxemic rats. The central administration of Mas receptor antagonist A779 (3 nmol in 2 µL, i.c.v.) abolished these anti-inflammatory effects in endotoxemic rats. Furthermore, Ang-(1-7) applied centrally restored mean arterial blood pressure (MABP) without affecting heart rate (HR) and prevented vascular hyporesponsiveness to norepinephrine (NE) and AVP in animals that received LPS. Together, our results indicate that Ang-(1-7) applied centrally promotes a systemic anti-inflammatory effect through the central Mas receptor and activation of the humoral pathway mediated by AVP.

## 1. Introduction

Endotoxemia, a classical model of systemic inflammation, is characterized by the amplified production of tumor necrosis factor alpha (TNF-α), interleukin-1β (IL-1β), interleukin-6 (IL-6), and nitric oxide (NO) by immune cells and vascular endothelium [1]. The overproduction of inflammatory mediators has been involved in hypotension, hyporesponsiveness to vasoactive agents, and alterations in the hypothalamic-neurohypophyseal axis during systemic inflammation [2,3]. Our previous studies showed a marked decrease in vasopressin (AVP) plasma levels and increase in activation of the hypothalamic-pituitary-adrenal (HPA) axis during the late phase of endotoxemia, observed 6 h after lipopolysaccharide (LPS) administration [4,5,6]. It has been demonstrated that NO may play a role on the regulation of the HPA axis and AVP synthesis in the hypothalamic paraventricular nucleus (PVN) during endotoxemia [7,8,9,10,11]. However, besides the immune stimulus, there are other ways to control the hypothalamic-neurohypophyseal axis, including osmolality, hypotensive stimulus, and activation of the renin-angiotensin system (RAS) [12].

It is well known that the RAS plays a key role in the modulation of many functions in the body. Angiotensin-(1-7) [Ang-(1-7)]/Mas receptor, the counter-regulatory axis of the RAS, exerts beneficial effects against the pathophysiologic conditions [13,14]. The expression of Mas receptor was observed in neurons, glia cells, and endothelial cells of cerebral vessels [15]. As the agonist for the Mas receptor, Ang-(1-7) is also produced in the brain, in areas including the hypothalamus [16]. In PVN, this peptide is a potent secretagogue of AVP and may participate in controlling its release by magnocellular neurons [17]. In addition to neuroendocrine actions, the activation of Ang-(1-7)/Mas receptor axis attenuates inflammation in several experimental models, including polymicrobial sepsis and cerebral ischemia [18,19,20]. In our most recent study, it has been shown that central administration of Ang-(1-7) prevented LPS-induced vascular hyporesponsiveness and hypotension due to an anti-inflammatory effect via activation of sympathetic signaling during the initial phase of endotoxemia [21]. However, the central mechanisms of Ang-(1-7) to control systemic inflammation in the late phase of endotoxemia are not well known. Considering that Ang-(1-7) acts as a central neuropeptide controlling AVP release and the HPA axis as well as the importance of these hormones for hypotensive and inflammatory response during endotoxemia [22,23,24,25], in the present study we aimed to determine whether Ang-(1-7) can modulate systemic inflammation through the activation of humoral pathways in late phase of endotoxemia.

## 2. Material and Methods

### 2.1. Animal Experiments

Experiments were performed on adult male Wistar rats (215–220 g) obtained from the animal facility of the University of São Paulo, Ribeirão Preto Campus. The animals were housed at a controlled temperature (24.0 ± 2 °C) and exposed to a daily 12 h light–dark cycle (lights on from 6:00 to 18:00 h) and provided with food and water ad libitum. All experimental protocols were performed in accordance with the guidelines of the Ethics Committee on Animal Experimentation of the Ribeirão Preto College of Nursing, University of São Paulo (CEUA Protocol 14.1.872.53.4).

### 2.2. Stereotaxic Surgery

Seven days before the experiment, the rats were anesthetized with a mixture of ketamine and xylazine (90 mg/kg and 9 mg/kg, respectively, i.p., diluted in 0.9% isotonic saline) (Aldrich, Milwaukee, WI, USA) and immobilized in a stereotaxic frame. A stainless steel guide cannula (0.4 mm) was introduced into the right lateral ventricle (coordinates: A: −1.6 mm, L: 1 mm, D: 3.6 mm from the bregma) [26]. The displacement of the meniscus in a water manometer ensured the correct position of the cannula into the lateral ventricle. The cannula was fixed to the skull with stainless steel screws and dental acrylic cement. A tight-fitting stylet was kept inside the guide cannula to prevent occlusion and infection. At the end of the surgery, all animals received an injection of a polyvalent veterinary pentabiotic (24.000 UI/kg; Zoetis, Brazil). The rats were allowed to recover for seven days.

### 2.3. Cannulation Procedures

For intravenous (i.v.) drug administration, the rats were anesthetized on the day before the experiment with ketamine and xylazine, and a flexible catheter (PE-10, Silastic^®^, Dow Corning CO., Midland, MI, USA) was inserted into the right internal jugular vein. For direct mean arterial blood pressure (MABP) and heart rate (HR) measurements, an additional catheter (PE-10 heat-sealed to PE-50) was inserted into the femoral artery. The catheters were tunneled under the skin and exteriorized in the back of the neck, as described previously [5]. The animals were housed separately and allowed to recover for 24 h before taking measurements.

### 2.4. Drug Administration

Drugs were administered to the conscious rats, alone or in combination, according to the following chronology: (a) The rats received an intracerebroventricular (i.c.v.) A779 (3 nmol in 2 µL) or saline (0.9%), a potent and selective antagonist for receptor of Ang-(l-7), the Mas receptor; (b) After 30 min, the rats received an i.c.v. injection of Ang-(1-7) (0.3 nmol in 2 µL) or saline (NaCl 0.9%); (c) 1 min after Ang-(1-7) injection the rats received an i.v. bolus LPS injection (1.5 mg/kg) or saline (0.9%). Control animals were injected with the same volume of saline through the same routes. The doses of Ang-(1-7), A779, and LPS used were based on previous studies from our group [21,27]. Thus, the rats were divided into eight experimental groups according to analysis: (1) Saline (i.c.v.) + Saline (i.c.v.) + Saline (i.v.) (control group); (2) Saline (i.c.v.) + Ang-(1-7) (i.c.v.) + Saline (i.v.); (3) Saline (i.c.v.) + Saline (i.c.v.) + LPS (i.v.); (4) Saline (i.c.v.) + Ang-(1-7) (i.c.v.) + LPS (i.v.); (5) A779 (i.c.v.) + Saline (i.c.v.) + Saline (i.v.); (6) A779 (i.c.v.) + Ang-(1-7) (i.c.v.) + Saline (i.v.); (7) A779 (i.c.v.) + Saline (i.c.v.) + LPS (i.v.); (8) A779 (i.c.v.) + Ang-(1-7) (i.c.v.) + LPS (i.v.).

### 2.5. Samples

Samples of PVN and supraoptic nucleus (SON) and blood were collected 6 h after LPS administration. After harvest, PVN and SON samples were stored in RNase-free microcentrifuge tubes in a freezer at −80 °C. A blood sample was collected with EDTA (1 mmol/L) and immediately centrifuged (3100 rpm, 4 °C, 15 min) to obtain plasma, which was stored in a freezer at −80 °C. Another blood sample was collected without anticoagulant and centrifuged (3500 rpm, 4 °C, 10 min) to obtain serum, which was also stored in a freezer at −80 °C.

### 2.6. Plasma Osmolality, Sodium, and Lactate Measurements

The determination of plasma osmolality was performed using an osmometer (Fiske OS Osmometer, Advanced Instruments, Norwood, MA, USA). Plasma sodium levels were analyzed using a quantitative electrode quantification technique (9180 Electrolyte Analyzer, Roche Diagnostics GmbH, Mannheim, Germany). To determine the lactate level, a commercial enzyme immunoassay kit (Quibasa Química Básica, Belo Horizonte, Brazil) was used.

### 2.7. Real-Time Polymerase Chain Reaction (RT-PCR)

After decapitation, PVN and SON samples were harvested, stored in RNase-free microcentrifuge tubes and frozen in liquid nitrogen within a 5-min time frame. Samples were then disintegrated in the presence of Trizol^®^ (Introvigen, Carlsbad, CA, USA), and RNA extraction followed the Trizol standard procedure with glicogen. RNA was quantified by spectrophotometry using nanodrop 1000 equipment (Thermo Fisher Scientific, Wilmington, DE, USA). The analysis of the genes of interest was performed by RT-PCR using TaqMan assays (*AVP* Rn00690189_g1; corticotropin-releasing hormone-*CRH* Rn01462137_m1; *TNF* Rn99999017_m1; *IL10* Rn01483988_g1; inducible nitric oxide synthase (*NOS2*) Rn00561646_m1; *GAPDH* Rn01775763_g1) as previously described in Faim et al. [28]. The results were expressed as normalized relative quantities (NRQ).

### 2.8. Corticosterone and Vasopressin (AVP) Measurements

The corticosterone and AVP radioimmunoassay were performed as previously described by Vecsei [29]. Plasma samples (25 μL) were extracted using ethanol, lyophilized, and stored at −20 °C until analysis of corticosterone. Plasma samples (0.5 mL) were extracted using the acetone/petroleum ether method, lyophilized, and stored at −20 °C until analysis of AVP. Assay sensitivity and intra- and inter-assay coefficients of variation were 0.4 μg/dL; 3.3% and 10.0% for corticosterone; and 0.7 pg/mL, 7.6%, and 12% for AVP. The results were expressed as μg/dL for corticosterone and pg/mL for AVP.

### 2.9. Cytokine Measurements

Levels of interleukin (IL)-1β (catalog # RLB00), IL-6 (catalog # R6000B), and interleukin-10 (IL-10) (catalog # R1000) were quantified by enzyme-linked immunosorbent assay (ELISA) using commercial kits from R&D Systems (Minneapolis, MN, USA) according to the user manual. The TNF level was quantified by an ELISA kit from Biolegend (catalog 438206) (San Diego, CA, USA) according to the user manual. The results were expressed as cytokine concentration in pg/mL based on standard curves.

### 2.10. Plasma Nitrite/Nitrate (NOx) Measurement

Plasma samples were deproteinized with 100 µL of absolute ethanol at 4 °C for 30 min. The samples then were centrifuged at 10,000 rpm for 5 min. After deproteinization, 5 µL of samples were injected into a reaction vessel containing vanadium trichloride. The NOx produced was detected as ozone-induced chemiluminescence using the Sievers Instruments NO analyzer (NOA model 280i; Boulder, Colorado). The results were expressed as NOx concentration in μM/L.

### 2.11. Thiobarbituric Acid Reactive Substance (TBARS) Measurement

Levels of TBARS were quantified by colorimetric assay using a commercial kit from Cayman chemical (#10009055, Michigan, MI, USA) according to the user manual. The results were expressed as TBARS concentration in nmol/L based on standard curve.

### 2.12. Mean Blood Pressure (MABP) and Heart Rate (HR) Measurements

On the day of the experiment, an arterial catheter was connected to a pressure transducer (TSD104A) and a data acquisition unit (MP100 System; BIOPAC Systems Inc, Santa Barbara, CA, USA) to record the MABP and HR of conscious and freely moving rats. The data were converted and analyzed using AcqKnowledge v.3.9.0 software (BIOPAC Systems Inc, Santa Barbara, CA, USA). A quiet environment was maintained to avoid stress, and the rats had pulsatile arterial pressure recorded at baseline conditions for 30 min. After LPS or saline injection, MABP and HR were then measured as a single time point at 360 min. The results were expressed as the difference from baseline.

### 2.13. Vascular Reactivity on Thoracic Aorta

After 6 h of LPS administration, the thoracic aorta of each animal of experimental groups (Saline (i.c.v.) + Saline (i.c.v.) + Saline (i.v.) (control group); (2) Saline (i.c.v.) + Ang-(1-7) (i.c.v.) + Saline (i.v.); (3) Saline (i.c.v.) + Saline (i.c.v.) + LPS (i.v.); (4) Saline (i.c.v.) + Ang-(1-7) (i.c.v.) + LPS (i.v.)) was sectioned and cut into four rings of the same size (4 mm) to normalize contractile forces. The rings were kept in two stainless steel stirrups and connected to an isometric force transducer (Letica Scientific Instruments, Barcelona, Spain) in a chamber containing Krebs solution (composition in mmol/L: NaCl 130.0; KCl 4.7; KH_2_PO_4_ 1.2; MgSO_4_ 1.2; NaHCO_3_ 14.9; C_6_H_12_O_6_ 5.5; CaCl_2_ 1.6), pH 7.4, supplied with a gas containing 95% O_2_ and 5% CO_2_ at 37 °C. Each ring was stretched to a resting tension of 1.5 g, which was maintained for 60 min for stabilization. In vitro, the rings were subsequently stimulated with phenylephrine (0.1 μmol/L), a selective α_1_-adrenergic receptor agonist, and the presence or absence of endothelium was verified using acetylcholine (1 μmol/L). Cumulative concentration-effect curves were constructed for norepinephrine (NE) (0.1 μmol/L–10 μmol/L) in the presence or absence of the aminoguanidine, NOS2 selective inhibitor (100 μmol/L), and AVP (1 nmol/L).

### 2.14. Statistical Analysis

Statistical analyses were performed using Prism 6.0 (GraphPad) software. Osmolality, sodium, lactate, cytokines, NOx, corticosterone, AVP, MABP, HR, and RT-PCR measurements were statistically analyzed by two-way ANOVA followed by the Bonferroni *post-hoc* test. The maximum constrictor effect (Emax) was considered as the maximal amplitude response reached in the concentration-effect curves for the contractile agent. The concentration of agents that produced half-maximal relaxation amplitude was determined after logit transformation of the normalized concentration-response curves and reported as negative logarithm (pD_2_) of the mean of individual values. The experimental sample n refers to the number of animals, and data are expressed as the means ± standard error of the mean (SEM). Differences were considered statistically significant when *p* < 0.05.

## 3. Results

### 3.1. Central Ang-(1-7) Attenuated the Lactate Level and Did Not Change Osmolality and Sodium Plasma Levels in Endotoxemia

LPS increased the lactate plasma level as compared with the control group (3.94 ± 0.15 *versus* 2.01 ± 0.28 mM; F = 20.34, *p* < 0.0001, respectively). Central administration of Ang-(1-7) attenuated the lactate plasma level in the presence of LPS (2.71 ± 0.22 *versus* 3.94 ± 0.15 mM; F = 0.0654, *p* < 0.05, respectively). The statistical analysis showed significant endotoxemia and treatment interaction (F = 6.92, *p* = 0.0133) for lactate. Neither central Ang-(1-7) nor LPS affected the plasma osmolality and levels of sodium (317.88 ± 5.95 and 311.90 ± 6.70 *versus* 298.75 ± 5.69 mOsm/Kg; 144.75 ± 0.75 and 144.60 ± 1.06 *versus* 144.33 ± 0.80 mEq/L, respectively) (Table 1).

### 3.2. Central Administration of Ang-(1-7) Did Not Attenuate Neuroinflammation in Endotoxemia

**Figure 1:**
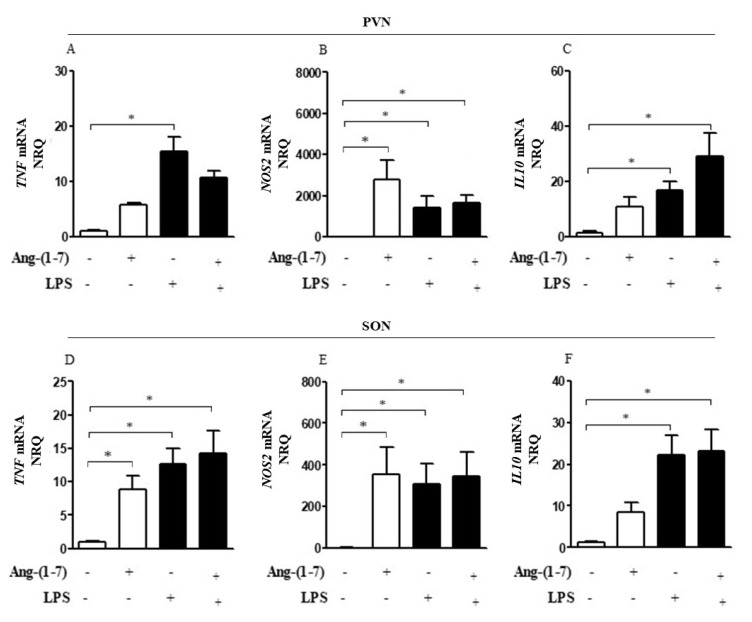
Central administration of Ang-(1-7) did not attenuate neuroinflammation in endotoxemia. Effect of central Ang-(1-7) on the *TNF* (**A**,**D**), *NOS2* (**B**,**E**), and *IL10* (**C**,**F**) gene expression in PVN and SON of rats 6 h after LPS administration (1.5 mg/Kg, i.v.). Ang-(1-7) (0.3 nmol in 2 µL, i.c.v.) was injected 1 min before LPS. Data are expressed as the mean ± SEM, *n* = 7–9. ANOVA, followed by Bonferroni *post-hoc* test. * *p* < 0.05 *versus* Saline (i.c.v.) + Saline (i.v.) group.

Neuroinflammatory analysis showed significant effects of LPS on *TNF*, *NOS2*, and *IL10* gene expression in PVN (F = 54.09, *p* < 0.0001; F = 6.69, *p* = 0.8169; F = 14.39, *p* = 0.0008, respectively) and SON (F = 11.34, *p* = 0.0022; F = 2.11, *p* = 0.1582; F = 20.86, *p* < 0.0001, respectively) (Figure 1A–F). Central administration of Ang-(1-7) did not change the *TNF*, *NOS2*, and *IL10* gene expression in the hypothalamic nuclei of endotoxemic rats, but there was a tendency to mitigate *TNF* and elevate *IL10* gene expression in PVN (F = 0.1492, *p* = 0.7025; F = 4.181, *p* = 0.0515, respectively) (Figure 1A,C).

### 3.3. Central Administration of Ang-(1-7) Attenuated Systemic Inflammation and Restored Plasma AVP Levels in Endotoxemic Rats via Mas Receptor

**Figure 2:**
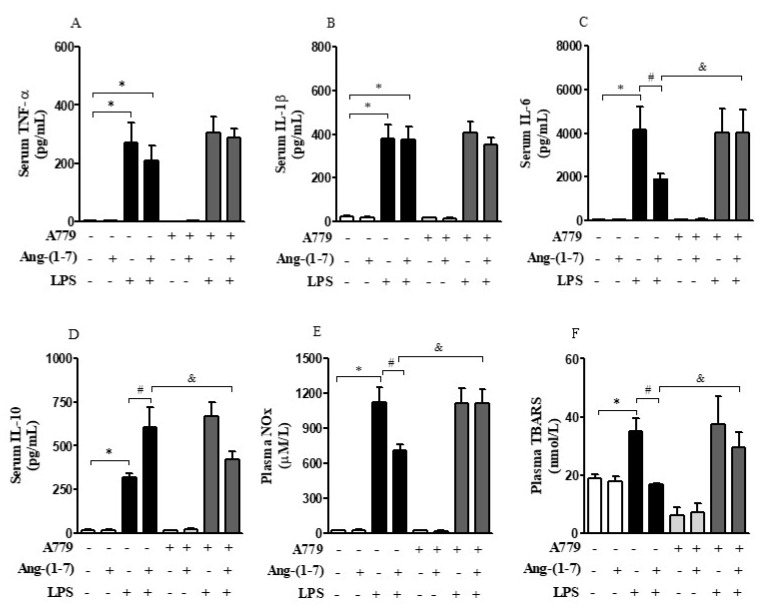
Central administration of Ang-(1-7) attenuated systemic inflammation in endotoxemic rats via Mas receptor. Effect of central Ang-(1-7) on the TNF-α (**A**), IL-1β (**B**), IL-6 (**C**), IL-10 (**D**), NOx (**E**), and TBARS (**F**) levels in rats 6 h after LPS administration (1.5 mg/Kg, i.v.). A779 (3 nmol in 2 µL, i.c.v.), a selective antagonist for Mas receptor, was injected 30 min before Ang-(1-7) administration (0.3 nmol in 2 µL, i.c.v.). Data are expressed as the mean ± SEM, *n* = 7–12. ANOVA, followed by Bonferroni *post-hoc* test. ^*^ *p* < 0.05 *versus* Saline (i.c.v.) + Saline (i.v.) group, ^#^ *p* < 0.05 *versus* Saline (i.c.v) + LPS (i.v.) group, and ^&^ *p* < 0.05 *versus* Ang-(1-7) (i.c.v) + LPS (i.v.) group.

LPS significantly increased the production of inflammatory markers analyzed including serum and plasma levels of TNF-α, IL-1β, IL-6, IL-10, NOx, and TBARS as compared with the control group (F = 70.12, *p* < 0.0001; F = 171.70, *p* < 0.0001; F = 60.02, *p* < 0.0001; F = 181.60, *p* < 0.0001; F = 297.90, *p* < 0.0001; F = 33.99, *p* < 0.0001, respectively). Central administration of Ang-(1-7) specifically decreased IL-6, NOx, and TBARS, and increased serum IL-10 levels in endotoxemic rats (F = 1.47, *p* < 0.05; F = 2.88, *p* = 0.0429; F = 22.75, *p* < 0.0001; F = 4.99, *p* = 0.0044, respectively); however, administration of A779 abrogated these peripheral anti-inflammatory responses (Figure 2A–F). Statistical analysis showed significant endotoxemia and central administration of Ang-(1-7) interaction (F = 1.43, *p* < 0.05; F = 5.18, *p* = 0.0036; F = 3.08, *p* = 0.0339; F = 16.55, *p* < 0.0001) for IL-6, IL-10, NOx, and TBARS levels, respectively.

**Figure 3:**
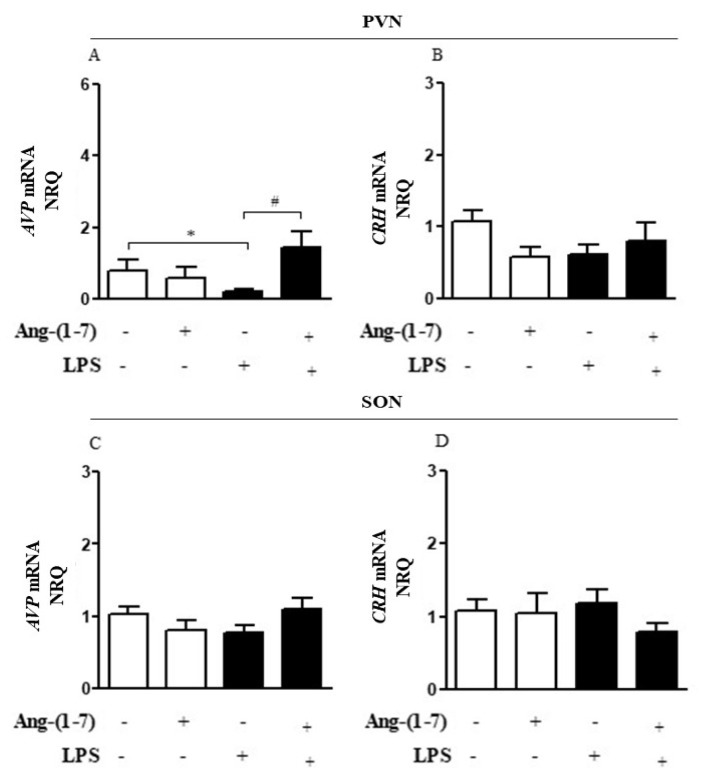
Central administration of Ang-(1-7) increased *AVP* gene expression in endotoxemic rats. Effect of central Ang-(1-7) on the *AVP* (**A**,**C**) and *CRH* (**B**,**D**) gene expression in PVN and SON of rats 6 h after LPS administration (1.5 mg/Kg, i.v.). Ang-(1-7) (0.3 nmol in 2 µL, i.c.v.) was injected 1 min before LPS. Data are expressed as the mean ± SEM, *n* = 7-9. ANOVA, followed by Bonferroni *post-hoc* test. ^*^ *p* < 0.05 *versus* Saline (i.c.v.) + Saline (i.v.) group, ^#^ *p* < 0.05 *versus* Saline (i.c.v) + LPS (i.v.) group.

**Figure 4:**
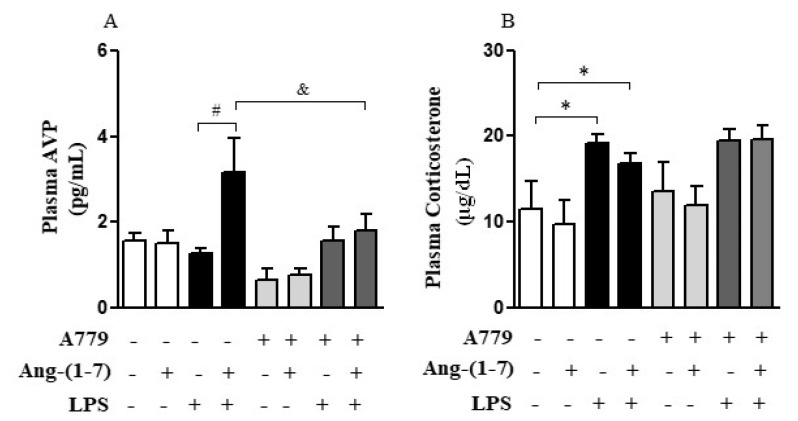
Central administration of Ang-(1-7) restored plasma AVP levels in endotoxemic rats via Mas receptor. Effect of central Ang-(1-7) on the AVP (**A**) and corticosterone (**B**) plasma levels in rats 6 h after LPS administration (1.5 mg/Kg, i.v.). A779 (3 nmol in 2 µL, i.c.v.), a selective antagonist for Mas receptor, was injected 30 min before central Ang-(1-7) administration (0.3 nmol in 2 µL, i.c.v.). Data are expressed as the mean ± SEM, *n* = 7–10. ANOVA, followed by Bonferroni *post-hoc* test. * *p* < 0.05 *versus* Saline (i.c.v.) + Saline (i.v.) group, ^#^ *p* < 0.05 *versus* Saline (i.c.v) + LPS (i.v.) group, and ^&^ *p* < 0.05 *versus* Ang-(1-7) (i.c.v) + LPS (i.v.) group.

Considering that the central nervous system (CNS) may activate neuroimmune pathways in order to control peripheral inflammation, we analyzed whether central administration of Ang-(1-7) affected the production of corticosterone or AVP (Figure 3A–D). Systemic administration of LPS decreased (F = 0.15, *p* < 0.05) *AVP* mRNA in PVN, and Ang-(1-7) prevented this decrease (F = 2.61, *p* < 0.05) in PVN. Statistical analysis showed significant endotoxemia and central administration of Ang-(1-7) interaction (F = 4.79, *p* = 0.0382) for *AVP* mRNA in PVN (Figure 3A). Moreover, central administration of Ang-(1-7) increased AVP plasma levels in endotoxemic rats (F = 3.90, *p* = 0.0130), and this effect was blocked by central injection of A779. Neither central Ang-(1-7) nor A779 administration affected the levels of corticosterone (F = 0.67, *p* = 0.5768) in the presence of LPS (Figure 4A,B).

### 3.4. Central Ang-(1-7) Restored Vascular Hyporesponsiveness and Prevented LPS-Induced Hypotension

**Figure 5:**
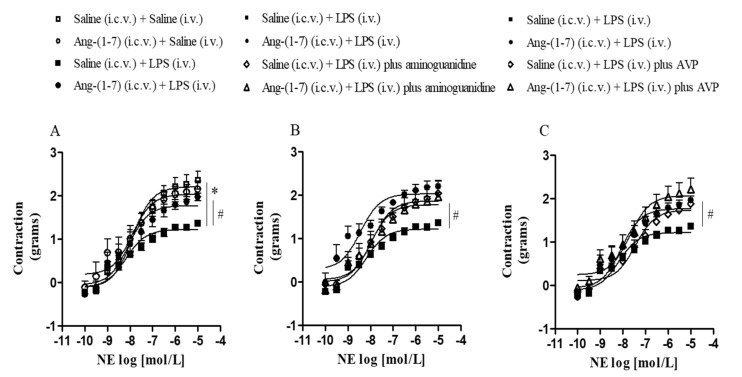
Central Ang-(1-7) restored vascular hyporesponsiveness in endotoxemic rats. Effect of central Ang-(1-7) (0.3 nmol in 2 µL, i.c.v.) in vascular reactivity of the thoracic aorta to NE in the absence (**A**) or presence of aminoguanidine, a NOS2 selective inhibitor (100 μmol/L) (**B**), and AVP (1 nmol/L) (**C**) in rats 6 h after LPS administration (1.5 mg/Kg, i.v.). Data are expressed as the mean ± SEM, *n* = 5–7. ANOVA, followed by Bonferroni *post-hoc* test. * *p* < 0.05 *versus* Saline (i.c.v.) + Saline (i.v.) group and ^#^ *p* < 0.05 *versus* Saline (i.c.v) + LPS (i.v.) group.

Systemic administration of LPS decreased the contractile response to NE in the Emax as compared with the control group, and central administration of Ang-(1-7) restored vascular hyporesponsiveness to NE in endotoxemic rats (Figure 5A and Table 2). The addition of aminoguanidine and AVP, in vitro, restored vascular hyporesponsiveness to NE induced by LPS (Figure 5B,C and Table 3 and Table 4). Thus, these results suggest that LPS effects on vascular function were dependent on the production of NO by NOS2 and reduction in the AVP plasma level during endotoxemia.

**Figure 6:**
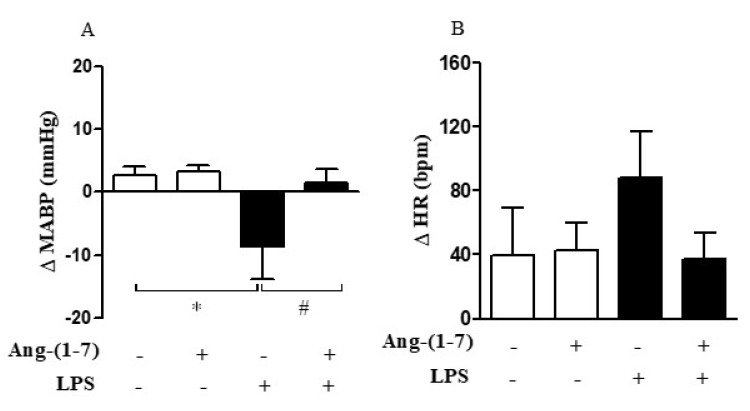
Central Ang-(1-7) prevented LPS-induced hypotension in endotoxemic rats. Effect of central Ang-(1-7) (0.3 nmol in 2 µL, i.c.v.) on mean arterial blood pressure (MABP) (**A**) and heart rate (HR) (**B**) in rats submitted to endotoxemia (LPS, 1.5 mg/Kg, i.v.) for a 6-h period. Data are expressed as the mean ± SEM, *n* = 6–9. ANOVA, followed by Bonferroni *post-hoc* test. * *p* < 0.05 *versus* Saline (i.c.v.) + Saline (i.v.) group, and ^#^ *p* < 0.05 *versus* Saline (i.c.v) + LPS (i.v.) group.

In our experimental model, LPS induced hypotension (F = 37.11, *p* < 0.0001) without promoting tachycardia (F = 0.76, *p* = 0.3935) (Figure 6A,B). Considering that the hyporesponsiveness to vasoconstrictor agents, such as AVP lead to vasoplegia and finally to hypotension, we analyzed whether Ang-(1-7) controls pressor response through the restoration of vascular responsiveness. Central administration of Ang-(1-7) prevented LPS-induced hypotension without affecting the HR (F = 15.20, *p* = 0.0010; F = 0.92, *p* = 0.3496, respectively) (Figure 6A,B). The statistical analysis showed significant endotoxemia and central administration of Ang-(1-7) interaction (F = 6.40, *p* = 0.0205) for MABP measurement.

## 4. Discussion

The main findings obtained in the present study were that Ang-(1-7) applied centrally prevented vascular hyporesponsiveness and hypotension by improving AVP impairment and systemic inflammation in endotoxemic rats. This effect is mediated by activation of Mas receptor located in the CNS, and it appears to be dependent on humoral pathway mediated by AVP.

The endotoxemia has been used as a powerful model to study the mechanisms involved in the pathophysiology of systemic inflammation [30]. During systemic inflammation, LPS activates peripheral immune cells causing synthesis and release of relatively high amounts of proinflammatory mediators. Furthermore, peripheral LPS challenge activates microglia, the major active immune cells in the CNS, leading to increased proinflammatory mediator levels in the brain, including elevated expression of TNF-α [31].

Evidence indicates that the RAS could promote pro-inflammatory effects within the hypothalamus, including microglial activation and production of pro-inflammatory mediators [32,33]. However, Ang-(1-7), a protective component of the RAS, exerts direct actions at the microglia to counteract these pro-inflammatory effects, via Mas receptor [34,35]. Mas receptor is expressed on neurons and there is evidence that this receptor is also expressed on microglia, astrocytes, and neurons, including the hypothalamus [15,19,35]. In the present study, we observed the elevated expression of TNF-α in PVN and SON during systemic inflammation, and central Ang-(1-7) administration showed a very clear tendency to promote anti-inflammatory effects particularly on PVN. Although it cannot be considered as being statistically significant, our results have the same anti-inflammatory profile as previous findings [13,19]. Although we haven’t found a potent central anti-inflammatory effect, we reported the systemic anti-inflammatory effect promoted by central Ang-(1-7), via Mas receptor, in endotoxemic rats.

Recent studies have reported elaborate neuroimmune interactions, in which the CNS controls the innate immune system and promotes efferent anti-inflammatory signals to regulate the excessive activation of the immune system. From a neuroimmune perspective, the CNS can regulate the activation of PVN resulting in the stimulation of humoral routes, mainly HPA axis activation and AVP release [36].

The activation of the HPA axis and secretion of corticosterone are clearly important in the control of inflammatory response during the late phase of endotoxemia [37]. Corticosterone exerts anti-inflammatory effects by inhibiting the function of nuclear factor kappa B and consequently modifying at both transcriptional and post-transcriptional levels of the pro-inflammatory genes on peripheral immune cells [38,39]. However, cytokines as well as NO production in hypothalamic nuclei are also critical for the activation of the HPA axis. Evidence has suggested that NO may be involved regulating the activity of the HPA axis, although it remains controversial as to whether NO has a stimulatory or an inhibitory effect on the release of CRH [10,40,41]. LPS-induced endotoxemia causes HPA axis activation through the parvocellular neurons activation that synthesize and secrete CRH in the hypothalamus [42]. Subsequently, CRH as well as AVP induces secretion of adrenocorticotropic hormone by the anterior pituitary and finally glucocorticoids from the adrenal cortex [43]. Our study corroborates the findings of previous studies. As expected, we observed an increase in plasma corticosterone levels after LPS treatment in rats, although no increase in CRH mRNA was observed in our model.

In addition, the increase in AVP mRNA induced by Ang-(1-7) did not potentiate the secretion of CRH, nor the plasma concentration of corticosterone in the rats in which LPS was administered. The use of A779 did not alter the plasma corticosterone level indicating that central Ang-(1-7) administration does not seem to participate in the activation of the HPA axis in our study. In accordance with our results, a recent study showed that ACE2 overexpression mice had no effect on plasma corticosterone under stress conditions [44]. Thus, our data suggest that other humoral pathways, such as AVP release, may mediate the anti-inflammatory induced by Ang-(1-7), in an experimental model of endotoxemia.

HPA axis activation is associated with peripheral anti-inflammatory effects of AVP, composing the humoral network for the control of systemic inflammation. During physiological conditions, the magnocellular neurons of PVN and SON in the hypothalamus synthesize and release AVP [45,46]. During endoxemia, the increase of plasma AVP levels was observed after LPS administration (early phase), followed by a rapid decrease over the next few hours, despite the presence of persisting hypotension [6,11]. In addition, no changes in the AVP stocks were seen in the neurohypophysis [7]. “AVP impairment” refers to the inappropriate decrease of AVP concentration seen in the late phase of a sepsis or endotoxemia model despite the persistent hypotension that can lead to shock and eventually to death [22]. In endotoxemia, AVP impairment in late phase of endotoxemia is associated with an increase in the synthesis of pro-inflammatory mediators during and the consequent late production of NO by NOS2 [47]. The data of the current study showed that the systemic administration of LPS decreased *AVP* mRNA in PVN and did not change the plasma AVP concentration even in the face of hypotension and exacerbated systemic inflammatory response, suggesting the occurrence of AVP impaired in our experimental model.

NO has been reported to participate in the modulation of AVP secretion; however, studies to date have produced contradictory evidence regarding the effect of NO in secretion of this hormone during systemic inflammation [11,39,48]. Ota et al. [49] and Yamaguchi, Watanabe, and Yamaya [50] showed that central injection of an NO donor caused an increase in plasma AVP concentration; whereas Carnio et al. [51] and Reis et al. [52] suggest that NO arising from NOS2 plays an important inhibitory role in AVP release during endotoxemia. The mechanism by which NO can modulate AVP release is not well established. It has been shown that NO can act directly or not inhibiting the postsynaptic activity of neurohypophysial neurons [53]. The data of the current study showed that Ang-(1-7) applied centrally has not reduced the increase in gene expression of *NOS2* in PVN and SON induced by systemic LPS. Interestingly, we observed that expression of NOS2 in PVN did not affect the Ang-(1-7) effect on AVP secretion, suggesting that NO showed no inhibitory effect in our experimental model. Similarly, Nomura et al. [9] did not observe any effect of the NOS2 gene disruption on AVP mRNA levels in the mouse hypothalamus. In addition, we also cannot exclude the possibility that the NOS2 posttranslational modifications have occurred, resulting in an impairment in NOS.

In this context, classic studies revealed that Ang-(1-7) induced AVP release in rat hypothalamus–pituitary explants with a potency equal to Ang II [17], whereas other studies indicated that central Ang-(1-7) administration did not change AVP release in basal conditions in rats [54]. In PVN, this peptide is a potent secretagogue of AVP and may participate in controlling its release by magnocellular neurons [17]. In fact, Qadri et al. [55] confirmed the first study performed in neurohypophysial explants by Schiavone et al. [16], showing that Ang-(1-7) microinjections into the PVN induce a release of AVP. The excitatory action of Ang-(l-7) on magnocellular neurons of the PVN provides evidence at a cellular level for a modulatory action of this heptapeptide on the regulation of vasopressin secretion [56]. Although these studies point to contrasting effects of Ang-(1-7) on the release of AVP, together they strengthen the concept that distinct Ang-(1-7) effects can be observed in specific brain areas depending on the existing pathophysiological condition [56]. Moriguchi et al. [57], evaluating the effect of i.c.v. Ang-(1-7) on the synthesis of AVP in the PVN of hypertensive rats, observed that the exogenous infusion of Ang-(1-7) proved to be as potent as to Ang II in stimulating AVP synthesis, reinforcing the role of this heptapeptide in the hydro-electrolytic control carried out by the PVN. Moreover, although there are classic studies demonstrating the effect of the release of AVP by Ang-(1-7), the role in which this heptapeptide promotes this action is not clear. Specifically in PVN, Ambuhl et al. [58] demonstrated that applications by Ang-(1-7) microiontophoresis resulted in an increase in the excitability of neurons in this region, this effect being blocked by A779. In our study, the pharmacological blockage of central Mas receptor blocked the rise in AVP plasma levels induced by central administration of Ang-(1-7) in endotoxemic rats. Considering the previously demonstrated expression of the Mas receptor in the hypothalamus [19], we suggest that Ang-(1-7) may have affected the electrical activity of the paraventricular magnocellular neurons to promote AVP secretion in our current study. However, we cannot exclude the possibility that other neuroimmune routes participate in Ang-(1-7) anti-inflammatory effects in our experimental model. Our group recently demonstrated that in early stage of endotoxemia, Ang-(1-7) elicits anti-inflammatory effects through the activation of a neuroimmune pathway involving central activation of Mas receptors and subsequent sympathetic autonomic signaling [21].

Although the number of studies demonstrating the systemic anti-inflammatory effects of AVP is still limited, AVP has been shown to have direct action on immune cells to control systemic inflammation. In cultured rat mesangial cells, AVP inhibits LPS- and IL-1β-stimulated NO and cGMP via V_1_ receptor [23], whereas in murine macrophages [25,59] AVP promotes anti-inflammatory effects by the inhibition of CD14 expression, endotoxin binding, and subsequent NF-κB activation. Regarding the peripheral anti-inflammatory effects of PVA, previous data highlighted the involvement of the V_2_ receptor in the reduction of sepsis-induced lung inflammation [60]. In the present study, we do not use AVP receptor antagonists as a pharmacological tool; however, we suggest that AVP may have mediated the attenuation of the systemic inflammatory response during endotoxemia by direct action on its receptors expressed in the cells of the immune system.

During endotoxemia, the vascular reactivity and endothelial barrier function are impaired, and this contributes to the hypotensive response [21]. In the vascular endothelium, LPS can cause synthesis and release of relatively high amounts of pro-inflammatory cytokines, which in turn produces relaxation of vascular smooth muscle tone resulting in hypotension and reduction of vasoconstrictor response to catecholamines [2,3]. IL-6 and NO are two well-recognized inflammatory mediators upregulated in inflammatory models, whereas IL-10 plays an immunoregulatory role inhibiting vascular IL-6 production [61]. Interestingly, IL-6 increases NOS2 activity in aortic smooth muscle cells and is associated with a drop in MABP in septic patients [62,63]. Previous studies of our group also showed that hypotension induced by LPS is dependent on NO release [4,5,6]. In this context, glucocorticoids and AVP also regulate different aspects of endothelial physiology during systemic inflammation. Glucocorticoids act as a negative regulator of NO and prostacyclin release in endothelial cells, whereas in the vascular smooth muscle cells, it increases arterial contractile sensitivity to NE and vascular resistance [64,65]. In respect to endogenous AVP, in addition to their anti-inflammatory property, it exerts a potent vasoconstrictor effect during systemic inflammation. In fact, concomitant glucocorticoid and AVP therapy may be associated with a survival benefit in septic patients [66]. The central Ang-(1-7) administration in our study attenuated hypotension and vascular hyporesponsiveness by reduction of NO production in endotoxemic rats. Herein, we reported that Ang-(1-7) reestablished vascular responsiveness to NE and AVP in the endotoxemia model. Based on these data, we speculate that the effect of Ang-(1-7) on vascular responsiveness is the result of the plasma reduction of IL-6, the systemic increase of IL-10, and the consequent decrease in NO production. Furthermore, Ang-(1-7) applied centrally has not been shown to have an effect on HR. Tachycardia, in turn, has been reported as a reflex compensatory response of hypotension and alterations can indicate reduction in the spontaneous baroreflex sensitivity during systemic inflammation [30]. In our experimental model, despite the tendency to increase the HR of endotoxemic animals, this response was not significant. Studies have already shown that Ang-(1-7) in the CNS improves baroreflex sensitivity [34,67]. Thus, although we did not observe the tachycardic response, we found an improvement in MABP accompanied by the restoration of HR in endotoxemic rats, suggesting a possible effect of Ang- (1-7) on baroreflex sensitivity. Moreover, hyperlactatemia is also used as an important biochemical marker of the progression of the systemic inflammation and the associated hypotension in endotoxemia models [6]. The improvement in vascular responsiveness, in the presence of AVP, may have led to the attenuation of the hypotensive response in endotoxemic animals treated with Ang-(1-7). Therefore, possibly the improvement of LPS-induced hypotension by Ang-(1-7) provides adequate perfusion in the endotoxemic rats, controlling hyperlactatemia in our study.

In conclusion, our data demonstrate the participation of central Ang-(1-7), via Mas receptor, on modulation of peripheral inflammation and on pressor response during endotoxemia. Although we have demonstrated the importance of the Ang-(1-7)/Mas receptor axis for the control of the inflammatory response in the endotoxemic animal, the present study has limitations. First, as in classic studies, we demonstrated the stimulating effect of the Ang-(1-7)/Mas receptor axis on the release of AVP during endotoxemia; however, we did not evaluate the exact mechanism by which this effect occurred in our study. Further studies are needed to investigate the exact mechanism by which central administration of Ang-(1-7) induces the synthesis and release of AVP in PVN in the experimental model of endotoxemia. Second, we did not use AVP receptor antagonists as a pharmacological tool in our study to assess the participation of AVP by mediating the peripheral effects of central Ang-(1-7) administration. Thus, mechanisms of these effects are not precisely elucidated, but our results suggest a strong participation of the humoral pathway mediated by AVP regulating the effects resulting from the Ang-(1-7) applied centrally.

## Figures and Tables

**Table 1:** Effect of central Ang-(1-7) administration in osmolality, sodium, and lactate plasma levels of the endotoxemic rats.

Experimental Groups	Plasma		
	Osmolality (mOsm/Kg)	Sodium (mEq/L)	Lactate (mM)
Saline (i.c.v.) + Saline (i.v.)	298.75 ± 5.69	144.33 ± 0.80	2.01 ± 0.28
Ang-(1-7) (i.c.v) + Saline (i.v.)	300.14 ± 4.38	144.50 ± 0.66	2.20 ± 0.42
Saline (i.c.v) + LPS (i.v.)	311.90 ± 6.99	143.60 ± 1.06	3.94 ± 0.15 *
Ang-(1-7) (i.c.v) + LPS (i.v.)	317.88 ± 5.94	144.75 ± 0.75	2.71 ± 0.22 ^#^

* *p* < 0.05 *versus* Saline (i.c.v.) + Saline (i.v.) group, ^#^ *p* < 0.05 *versus* Saline (i.c.v) + LPS (i.v.) group. ANOVA, followed by Bonferroni *post-hoc* test.

**Table 2:** Values of maximum constrictor effect (Emax) and pD_2_ obtained from concentration-response curves in response to NE in thoracic aorta rings of control or endotoxemic rats treated or not with Ang-(1-7).

Experimental Groups	NE		
	*n*	Emax	pD_2_
Saline (i.c.v.) + Saline (i.v.)	7	2.36 ± 0.20	7.86 ± 0.09
Ang-(1-7) (i.c.v) + Saline (i.v.)	6	2.16 ± 0.19	8.07 ± 0.16
Saline (i.c.v) + LPS (i.v.)	6	1.36 ± 0.06 *	8.09 ± 0.09
Ang-(1-7) (i.c.v) + LPS (i.v.)	7	1.97 ± 0.09 ^#^	8.05 ± 0.12

* *p* < 0.05 *versus* Saline (i.c.v.) + Saline (i.v.) group, ^#^ *p* < 0.05 *versus* Saline (i.c.v) + LPS (i.v.) group. ANOVA, followed by Bonferroni *post-hoc* test.

**Table 3:** Values of Emax and pD_2_ obtained from concentration-response curves in response to NE, in the presence or absence of aminoguanidine (in vitro), in thoracic aorta rings of endotoxemic rats treated or not with Ang-(1-7).

Experimental Groups	Aminoguanidine		
	*n*	Emax	pD_2_
Saline (i.c.v.) + LPS (i.v.)	5	1.99 ± 0.12	8.09 ± 0.09
Ang-(1-7) (i.c.v) + LPS (i.v.)	5	2.57 ± 0.53 ^#^	8.01 ± 0.12
Saline (i.c.v) + LPS (i.v.) *plus* Aminoguanidine	5	2.20 ± 0.14 ^#^	7.84 ± 0.13 ^#^
Ang-(1-7) (i.c.v) + LPS (i.v.) *plus* Aminoguanidine	7	2.04 ± 0.29	7.97 ± 0.12

^#^*p* < 0.05 *versus* Saline (i.c.v) + LPS (i.v.) group. ANOVA, followed by Bonferroni *post-hoc* test.

**Table 4:** Values of Emax and pD_2_ obtained from concentration-response curves in response to NE, in the presence or absence of AVP (in vitro), in thoracic aorta rings of endotoxemic rats treated or not with Ang-(1-7).

Experimental Groups	AVP		
	*n*	Emax	pD_2_
Saline (i.c.v.) + LPS (i.v.)	6	1.36 ± 0.06	8.09 ± 0.08
Ang-(1-7) (i.c.v.) + LPS (i.v.)	5	2.03 ± 0.07 ^#^	8.05 ± 0.12
Saline (i.c.v.) + LPS (i.v.) *plus* AVP	5	1.90 ± 0.17 ^#^	7.60 ± 0.11 ^#^
Ang-(1-7) (i.c.v.) + LPS (i.v.) *plus* AVP	6	2.22 ± 0.25	7.83 ± 0.14

^#^*p* < 0.05 *versus* Saline (i.c.v) + LPS (i.v.) group. ANOVA, followed by Bonferroni *post-hoc* test.

## Data Availability

We included individual values in each figure which show all the data in the paper itself. The raw data supporting the findings of this manuscript will be provided by the authors at any time to the reviewers and thereafter to any researcher after publication in Cells.
